# Intestinal metabolomics in premature infants with late-onset sepsis

**DOI:** 10.1038/s41598-024-55398-7

**Published:** 2024-02-26

**Authors:** Jingfei Liu, Li Zhang, Dong Li, Xiaotong Yu, Ying Gao, Ying Zhou

**Affiliations:** 1 Department of Neonatology, Dalian Women and Children’s Medical Group, Dalian, 116037 China; 2 Department of Neonatology, The Second Affiliated Hospital of Dalian Medical University, Dalian, 116027 China; 3 Department of Neonatology, The First Affiliated Hospital of Dalian Medical University, Dalian, 116011 China

**Keywords:** Biomarkers, Diseases, Medical research

## Abstract

We aimed to investigate the characteristics of intestinal metabolomics and non-invasive biomarkers for early diagnosis of late-onset sepsis (LOS) by analyzing gut metabolites in preterm infants with LOS. We collected stool samples from septic and healthy preterm infants for analysis by liquid chromatography–mass spectrometry. 123 different metabolites were identified and 13 pathways were mainly involved. Glycine, serine, and threonine metabolism; glyoxylate and dicarboxylic acid metabolism; glutathione metabolism; primary bile acid biosynthesis; steroid synthesis; pentose and glucuronic acid interconversion may be involved in the pathogenesis of LOS in preterm infants. The significant changes of N-Methyldopamine, cellulose, glycine, gamma-Glutamyltryptophan, N-Ribosylnicotinamide and 1alpha, 25-dihydroxycholecalciferol showed specific diagnostic values and as non-invasive biomarkers for LOS.

## Introduction

Neonatal bacterial sepsis is associated with significant systemic inflammation, the development of multiple organ failure, and potentially life-threatening complications in neonates^1^. The prevalence of late-onset sepsis (LOS) in premature babies ranges from 20 to 30%^2^, with an associated mortality rate of approximately 15%. The prevalence of gestational age (GA) ranging from 25 to 28 weeks is 21%, while the occurrence of birth weight (BW) falling within the range of 751 to 1000 g is 28%^3^. The clinical signs of LOS are not well-defined, and the gold standard for diagnosing this condition remains blood culture^4^. Treatment with empiric antibiotics, blood sample volume, and other factors significantly decreased the percentage of proven blood cultures^5^, and sepsis in patients with blood culture-proven is only 30–40%^6^. However, a noninvasive, prompt diagnosis of LOS using a sensitive biomarker would benefit premature infants who are vulnerable to the pain and anemia associated with frequent blood tests.

The advent of novel technologies, including metabolomics, genomics, and proteomics, has presented a promising avenue for investigating neonatal sepsis. The utilization of metabolomics for the early detection and monitoring of disease progression presents significant benefits, as changes in metabolite profiles can provide insights into the host's specific pathological physiology^7^. By contrast, the metabolite serves as a direct biomarker of biochemical activity, with its biological effects being more closely associated with gene phenotypes^8^. The application of metabolomics has been extensively employed in the investigation of adult disorders, encompassing the areas of disease prevention, diagnosis, and particularly in cases where the etiology is unknown or in critical sickness conditions. Concurrently, there is a deficiency of research in the field of neonatology^9^.

Intestinal microbiota can produce a significant variety of metabolites through different pathways involved in regulating intestinal mucosal barrier function and affecting the immune system^10^. Microbes and their metabolites can stimulate macrophage function and block the progression of infectious diseases^11^. Microbial metabolites are considered critical mediators of the response to extrinsic organ injury, and there are relatively few studies on the effects of microbial metabolites on the progression of sepsis in preterm infants. Therefore, research on microbial metabolites may provide a new direction for the diagnosis of neonatal sepsis^12^, and regulation of gut metabolites to maintain intestinal immune balance, mitigating the occurrence of sepsis has promise as a prospective therapeutic approach.

## Methods

### Baseline of the subjects

Newborn preterm infants admitted to the hospital from January 2022 to August 2023 were recruited as study subjects. Clinical information (including general information, laboratory examination, and perinatal conditions) was recorded from admission, and they were divided into LOS group and control group according to diagnosis. Two groups of subjects were matched for gender, GA, BW, and all participants were singletons. During the observation period, none of the patients received intensive phototherapy or exchange transfusion for hyperbilirubinemia; all preterm infants were fed mainly with preterm formula (88 kcal/100 ml). Details are given in Table 1. The hospital ethics committee approved the study (No. 2022034), and we had obtained the informed consents from all subjects’ legal guardians.

**Table 1:** Baseline characteristics of subjects.

Baseline characteristic	LOS group (n = 18)	Control group (n = 42)	*P* value (Bilateral)
Maternal			
Age (years)	31.5 (4.8)	33.0 (6.0)	0.445^∆^
Cesarean section (%)	9 (50.0)	21 (50.0)	1.000
Gestational hypertension (%)	3 (16.7)	7 (16.7)	1.000
GDM (%)	4 (22.2)	4 (9.5)	0.362
PROM > 18 h (%)	8 (44.4)	17 (40.5)	0.775
HCA (%)	6 (33.3)	10 (23.8)	0.656
ACS (%)	17 (94.4)	35 (83.3)	0.456
Neonatal			
Male (%)	9 (50.0)	24 (57.1)	0.610
GA (w)	29.2 (1.9)	30.2 (1.6)	0.057^∆^
BW (g)	1407.5 (371.3)	1430.0 (475.0)	0.528^∆^
Enteral feeding in 24 h (%)	18 (100.0)	39 (92.9)	0.605
Pulmonary surfactant (%)	3 (16.7)	11 (40.5)	0.073
Invasive mechanical ventilation time (d)	7.5 (10.5)	2.0 (9.3)	0.215^∆^
Oral probiotics (%)	3 (16.7)	4 (9.5)	0.736
Blood transfusion (%)	11 (61.1)	30 (71.4)	0.431

*GDM* gestational diabetes mellitus, *PROM* premature rupture of membrane, *HCA* histologic chorioamnionitis, *ACS* antenatal corticosteroids, *GA* Gestational age, *BW* birth weight, ∆ Presents the Mann–Whitney U test results, and others present chi-square test results.

*p* < 0.05 indicates statistically significant differences between groups.

### Patient recruitment

*Inclusion criteria* GA < 32w and singleton.

*Exclusion criteria* (1) Early-onset sepsis (EOS): infants diagnosed with sepsis within 72 h after birth; (2) Severe congenital intestinal malformation (congenital intestinal atresia, anal atresia, intestinal perforation); (3) Newborns with congenital or suspected congenital metabolic diseases; (4) Prenatal diagnosis of chromosomal abnormalities; (5) Mothers with inherited metabolic diseases histories, or intrahepatic cholestasis of pregnancy (ICP) and abnormal liver function during pregnancy. All the above diagnoses were performed following guidelines.

*LOS diagnostic criteria*^13^ (1) Suspected diagnosis: > 3 days of age with abnormal clinical manifestations. If no abnormal clinical manifestations, the negative blood culture, and two consecutive blood non-specific tests with an interval of 24 h are less than two positive items, sepsis can be excluded. (2) Clinical diagnosis of abnormalities: meeting any of the following conditions: ① blood non-specific examination ≥ 2 positive items; ② cerebrospinal fluid laboratory tests meet purulent meningitis changes; ③ pathogenic bacteria DNA detected in the blood. (3) Confirmed diagnosis: infants with clinical manifestations and the positive blood culture or cerebrospinal fluid (or other sterile cavity fluid) culture were proven.

### Sample collection and preparation

**Figure 1:**
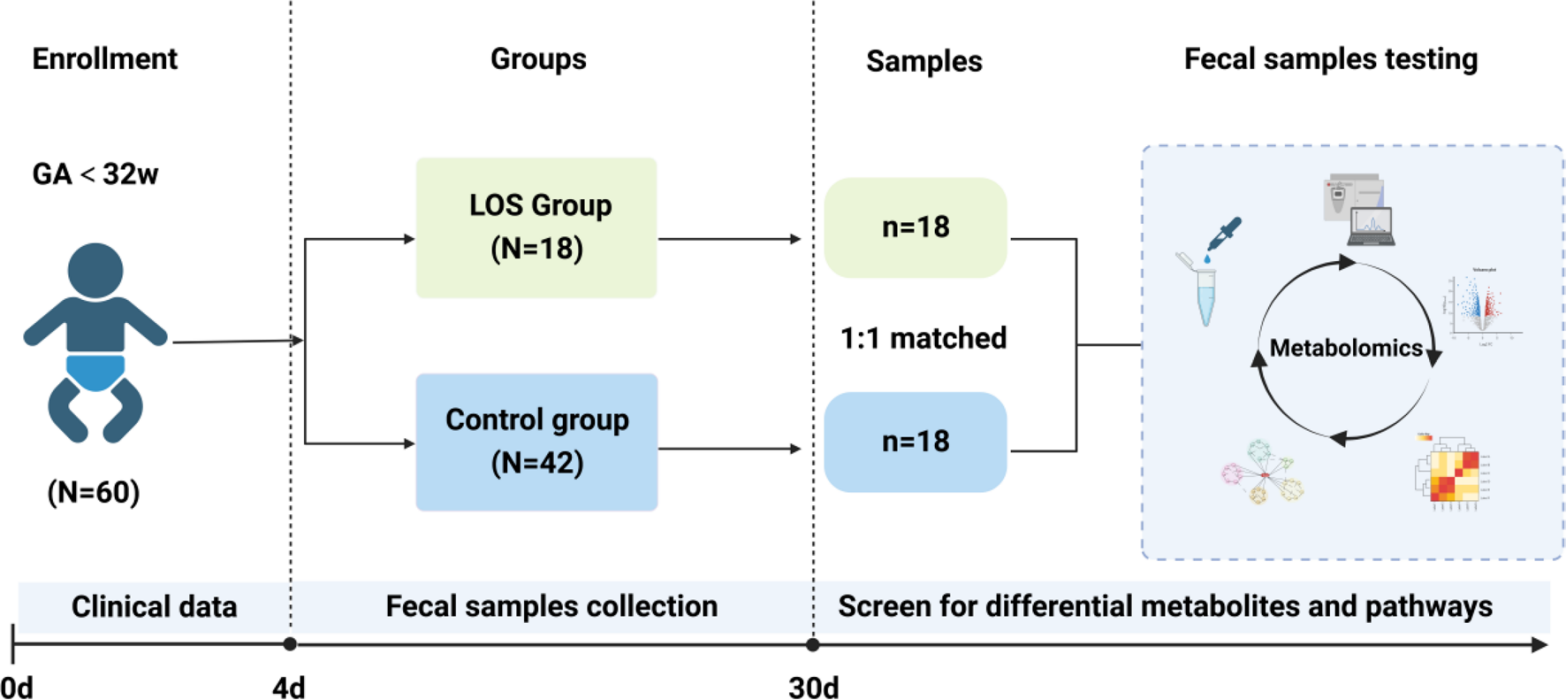
Fecal samples collection process diagram.

From 4 to 30 days after birth, fecal samples (at least 100–200 mg each time) were collected once a day (8–11 a.m.) and deposited in a sterile frozen storage tube. Sample contamination was prevented during the collection process, and samples were stored in a refrigerator at − 80 °C within 2 h. If the infant did not defecate, it remained for the next day. The fecal samples from the LOS (the onset day) and control groups were subjected to untargeted metabolomics analysis. The flow chart of fecal sample collection in preterm infants is shown in Fig. 1.

### LC–MS analysis

Untargeted metabolomics was performed using mass spectrometry and liquid chromatography-mass spectrometry (LC–MS) to collect metabolite profiles of samples, and we obtained m/z and retention time of compounds as putative metabolites. Both positive and negative ion modes were utilized to improve metabolite coverage. The original mass spectrum data was preprocessed, extracting peak list information and correcting the data. Statistical analyses were conducted to identify differentially expressed metabolites under different treatment conditions. Finally, the metabolites and differential metabolites were categorized, and pathway analysis was performed.

Specific methods for metabolite extraction were as follows: Weigh 25 mg sample into 1.5 ml EP tube accurately and add 800 µL pre-cryoprecipitate (methanol: acetonitrile: pure water = 2:2:1). Add two small steel balls, grind in the mill (60 Hz, 4 min), the sample after taking out the steel ball ice bath ultrasonic 10 min (power 80 Hz) and refrigerate at − 20 °C for 120 min. Centrifuge for 15 min (25,000 g, 4 °C), take 600µL supernatant and repeat once, and drain the supernatant in a freeze-draining machine. The supernatant was obtained by adding 600µL of 10% methanol solution to the ice bath for 10 min (power 80HZ) and centrifuging for 15 min (25000 g, 4 °C). Take 50µL of each sample and mix into a QC.

### Statistical analysis

GraphPad Prism (version 9.5.1) (https://www.graphpad.com/support/) was used to analyze the data, and Microsoft Excel (2007) was used to organize and manipulate the data. Non-parametric data were tested by Mann–Whitney U test and presented by median and interquartile range (IQR). Counting data are expressed as cases or as percentages N (%), using chi-square test or Fisher exact probability analysis. *P* value < 0.05 was considered statistically significant.

In our study, the Xevo G2-XS QTOF mass spectrometer (Waters, UK) was used for the acquisition of mass spectral data. Data was preprocessed using the meta-X ^14^, including missing value filling, mass ion removal, and correction with the (Quality control based robust LOESS signal correction, QC-RSC) method. Finally, filter out the ions with a relative standard deviation (RSD) > 30% in all QC samples. Progenesis QI (version 2.2) and the R package meta-X^14^ were used for univariate and multivariate analysis of mass spectra data to obtain differential metabolites between groups. Methods include parametric and non-parametric tests, differential expression multiple analysis and principal component analysis (PCA). The variable importance in projection (VIP) of the first two principal components of the multivariate Orthogonal Partial Least Squares-Discriminant Analysis (OPLS-DA) model was used to screen the differentially expressed metabolites by univariate analysis of fold-change and *p *value. Screening criteria: (1) VIP ≥ 1; (2) fold-change ≥ 1.2 or ≤ 0.8333; (3) *p* value < 0.05. Pathways were screened using Kyoto Gene and Kyoto Encyclopedia (KEGG)^15^, Human Metabolome Database (HMDB) and Lipidmaps. R for Windows (version 4.2.2) and metaboAnalyst 5.0 (https://www.metaboanalyst.ca) were used for data visualization analysis.

### Ethical approval

The authors are responsible for all parts of the work. All methods were carried out in accordance with relevant guidelines and regulations. This study was done in line with the Helsinki Declaration (as revised in 2013). The experimental protocols were approved by the Dalian Women and Children’s Medical Group Ethics Committee (No. 2022034). We confirm that this study was informed consent by all subjects’ legal guardians.

## Results

### Maternal and neonatal characteristics

The study included 60 preterm infants: GA was (29.85 ± 1.25) w and BW was (1422.50 ± 254.05)g. All infants were born in the same obstetric unit and transferred to the NICU immediately after birth for treatment. Maternal baseline characteristics were comparable between the two groups (*P* > 0.05), including age, cesarean delivery, gestational hypertension, gestational diabetes mellitus, preterm rupture of membranes > 18 h, and histological chorioamnionitis. The neonates were also comparable in terms of clinical characteristics (*P* > 0.05), including gender, GA, BW, small for gestational age (SGA), duration of mechanical ventilation, 24-h enteral feeding (all received mixed feeding due to early breast milk insufficiency), pulmonary surfactant supplementation and preterm probiotics (Table 1).

### Onset days, primary symptoms, and blood culture results of LOS infants

In this study, the average age of onset of LOS was (16.22 ± 6.23) d. The main clinical symptoms of 18 infants with LOS included apnea occurred in 7 cases; hypotonia, poor condition and irritability in 5 cases; respiratory distress in 3 cases; arterial hypotension and/or poor perfusion occurred in 2 cases; abdominal distension in 1 case. Blood cultures were positive in 18 of 60 infants (30%). Enterococcus faecalis was identified in 6 cases, Escherichia coli in 5 cases, Staphylococcus epidermidis in 4 cases, Staphylococcus haemolyticus in 1 case, Candida glabrata in 1 case, and Acinetobacter baumannii in 1 case.

### LC–MS results

#### Total fecal metabolites in preterm infants

18 fecal samples from the LOS group and 18 fecal samples from control group (1:1 matched) were subjected to untargeted metabolomics analysis, and there is no statistically significant difference in clinical information between the two groups of infants. LC–MS was used to collect the metabolite profiles of the samples and the raw mass spectrometry data were pre-processed to extract the peak list information and the data were corrected. The QC sample is used to balance the chromatography-mass spectrometry system prior to sample detection and is used to evaluate the stability of the mass spectrometry system during sample detection (Figure S1). The molecular weight, retention time, peak area and identification results were further determined and annotated using KEGG database, Lipid maps database and HMDB database. Finally, pathway annotation was performed to classify the obtained metabolites and identify their metabolic pathways.

**Figure 2:**
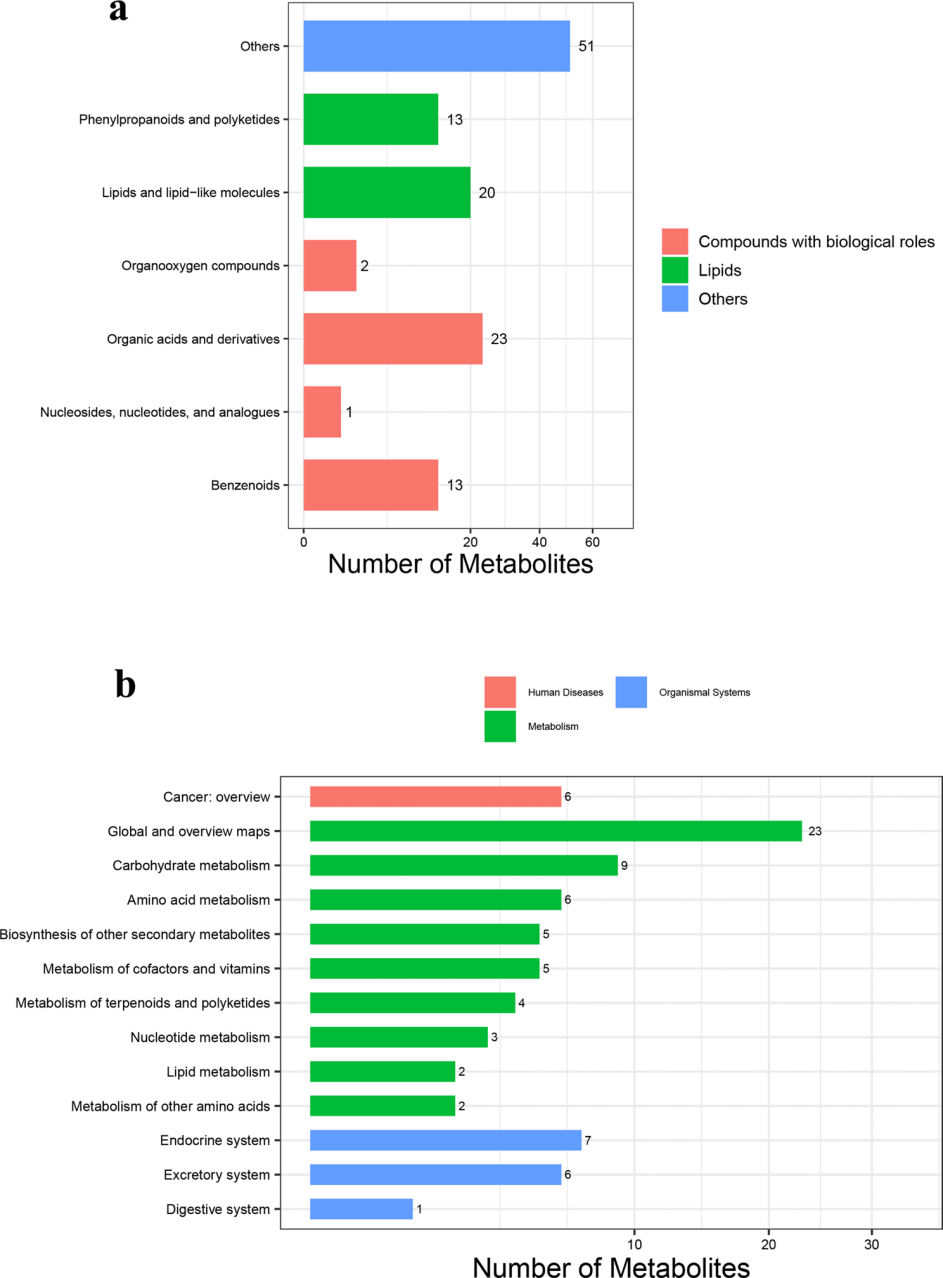
Identification of total metabolites and metabolic pathways. (**a**) Identification of metabolites. The abscissa is the number of metabolites, the ordinate is the type of metabolites, and the different colors represent the biological role of metabolites. (**b**) Identification of metabolic pathways. The different colors represent the different types of metabolic pathways in which the metabolites are involved, the abscissa represents the number of metabolites, and the ordinate represents the biological functions they exert.

A total of 123 different metabolites were identified, including 23 organic acids and derivatives, 13 benzenoids, 20 lipids and lipid-like molecules, 2 organooxygen compounds, 1 nucleoside or nucleotide analogue and 51 others (Fig. 2a). Thirteen metabolic pathways are mainly involved: global and overview mapping, glucose metabolism, amino acid metabolism, metabolism of cofactors and vitamins, metabolism of terpenoids and polyketides, nucleotide metabolism, lipid metabolism, endocrine system, excretory system and digestive system (Fig. 2b).

### Multivariate statistical analysis of fecal metabolites

**Figure 3:**
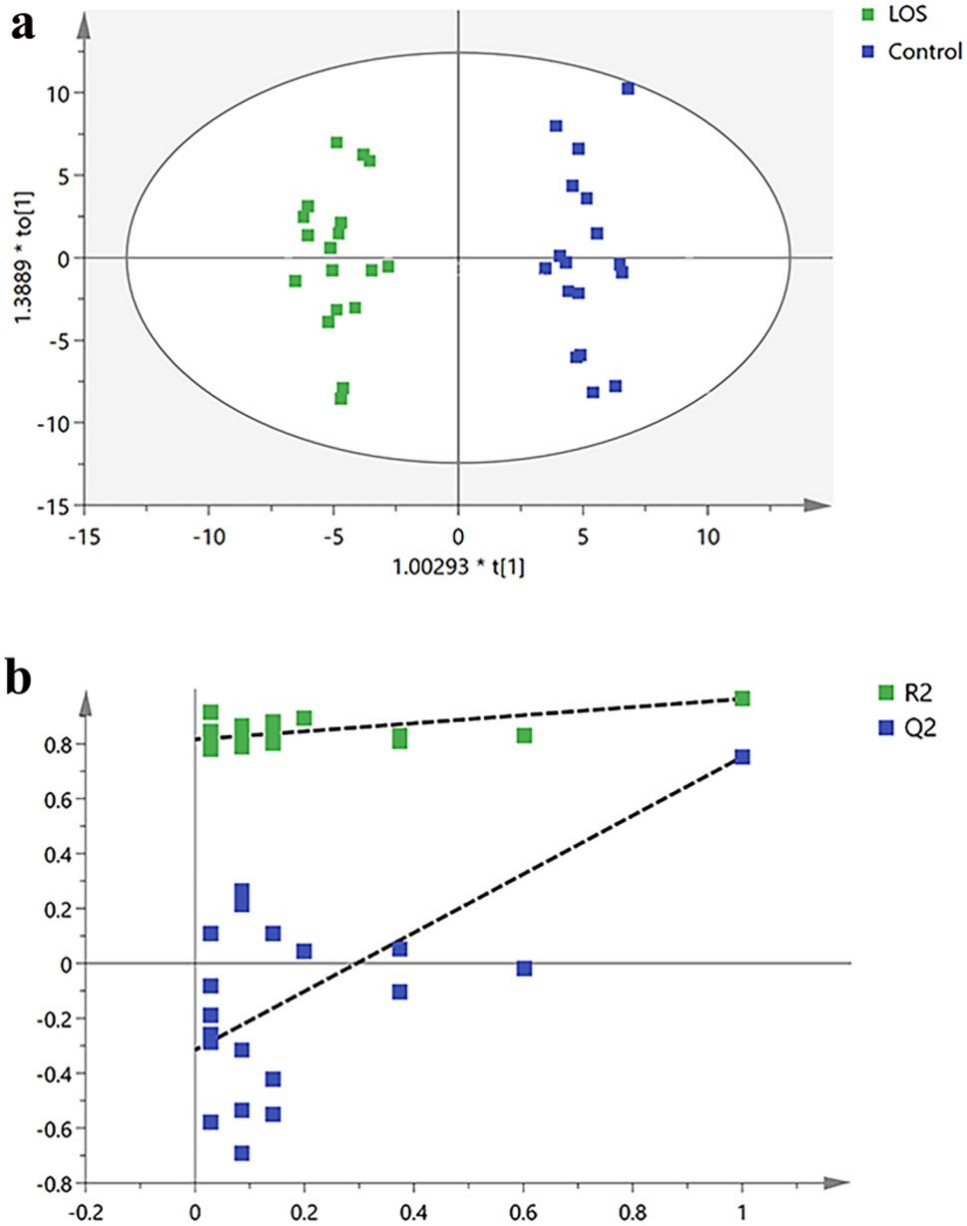
The score plot of the OPLS-DA model. (**a**) The OPLS-DA score map. Green points represent the LOS group, and blue points represent the control group. (**b**) Permutation test. Green boxes represent the R^2^ value and blue boxes represent the Q^2^ value.

To accurately identify the differential metabolites in the feces of LOS preterm and non-infected preterm infants, the total peak area normalized the metabolic data of the fecal samples of the two groups, and the samples were further analyzed using the OPLS-DA model. The OPLS-DA score plot proved that the samples of the two groups were significantly separated (Fig. 3a), and 200 permutation tests were performed (Fig. 3b). In the permutation test model, R^2^ = 0.81, Q^2^ = − 0.335, and the intercept of the Q^2^ regression line on the ordinate was less than 0, indicating that there was no overfitting and that the OPLS-DA model was reliable.

### Screening of differential metabolites in preterm feces between groups

**Figure 4:**
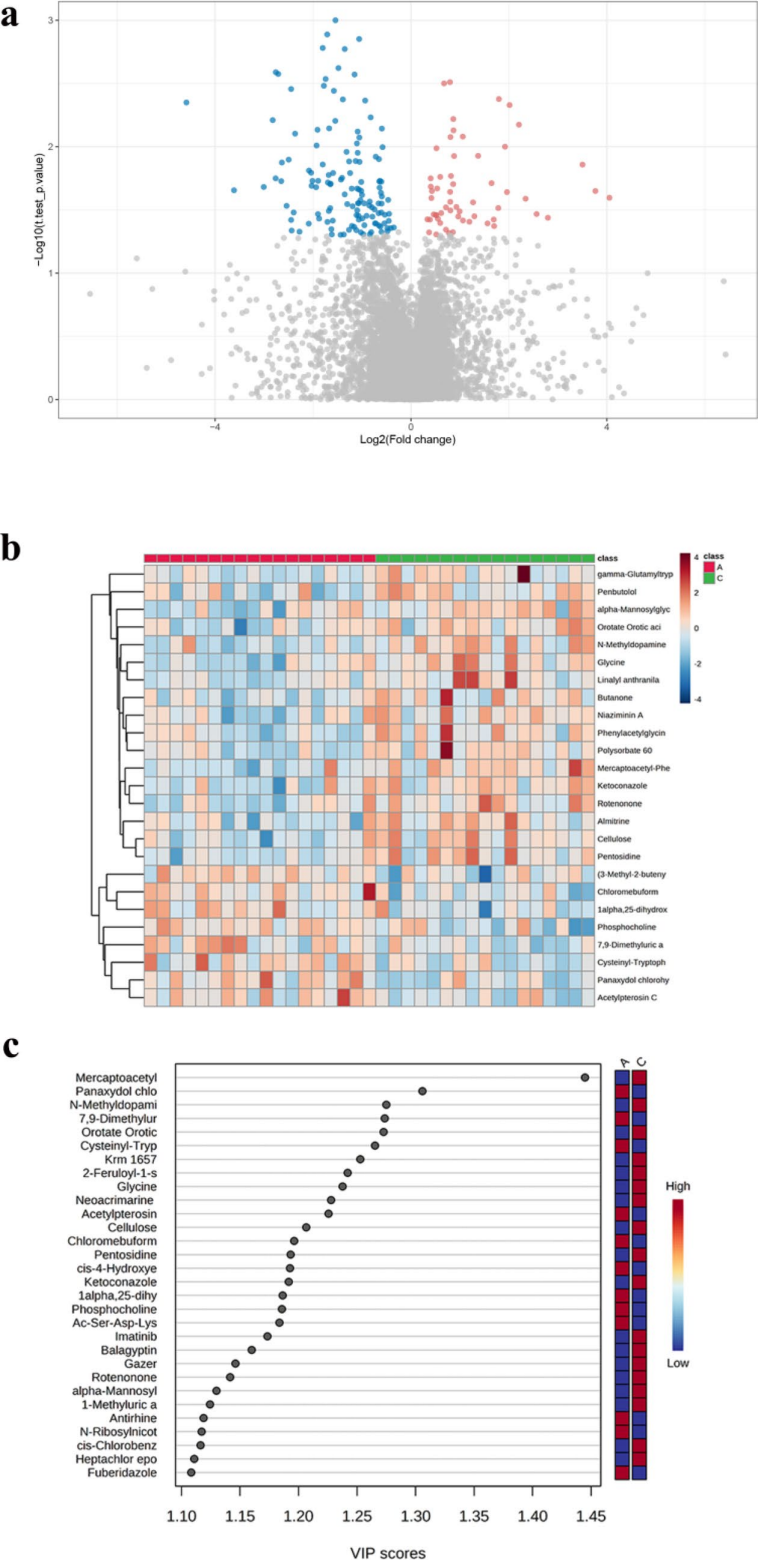
Screening of differential metabolites. (**a**) The volcano map. Red represents up-regulated metabolites, and blue represents down-regulated metabolites. (**b**) Heat map. Each column represents a sample with different colors indicating different intensities, with colors from blue to red indicating low to high intensities. (**c**) First 30 VIP score plot. A represents the LOS group, and C represents the control group.

T-test and Fold change analysis (FC analysis) were used to screen differential metabolites. The criteria for screening differential metabolites were VIP (variable importance in projection) ≥ 1, Log2 FC ≥ 1.2 or < 0.8333, and *p *value < 0.05. A total of 186 differential metabolites were identified in this study, of which 57 were up-regulated, and 129 were down-regulated. Volcano plots and heat maps show differences in these metabolites between the LOS and control groups (Fig. 4a, b). The VIP score plot shows the differential metabolites with VIP scores ≥ 1 for the First 30 (Fig. 4c).

### ROC analysis of metabolites

**Figure 5:**
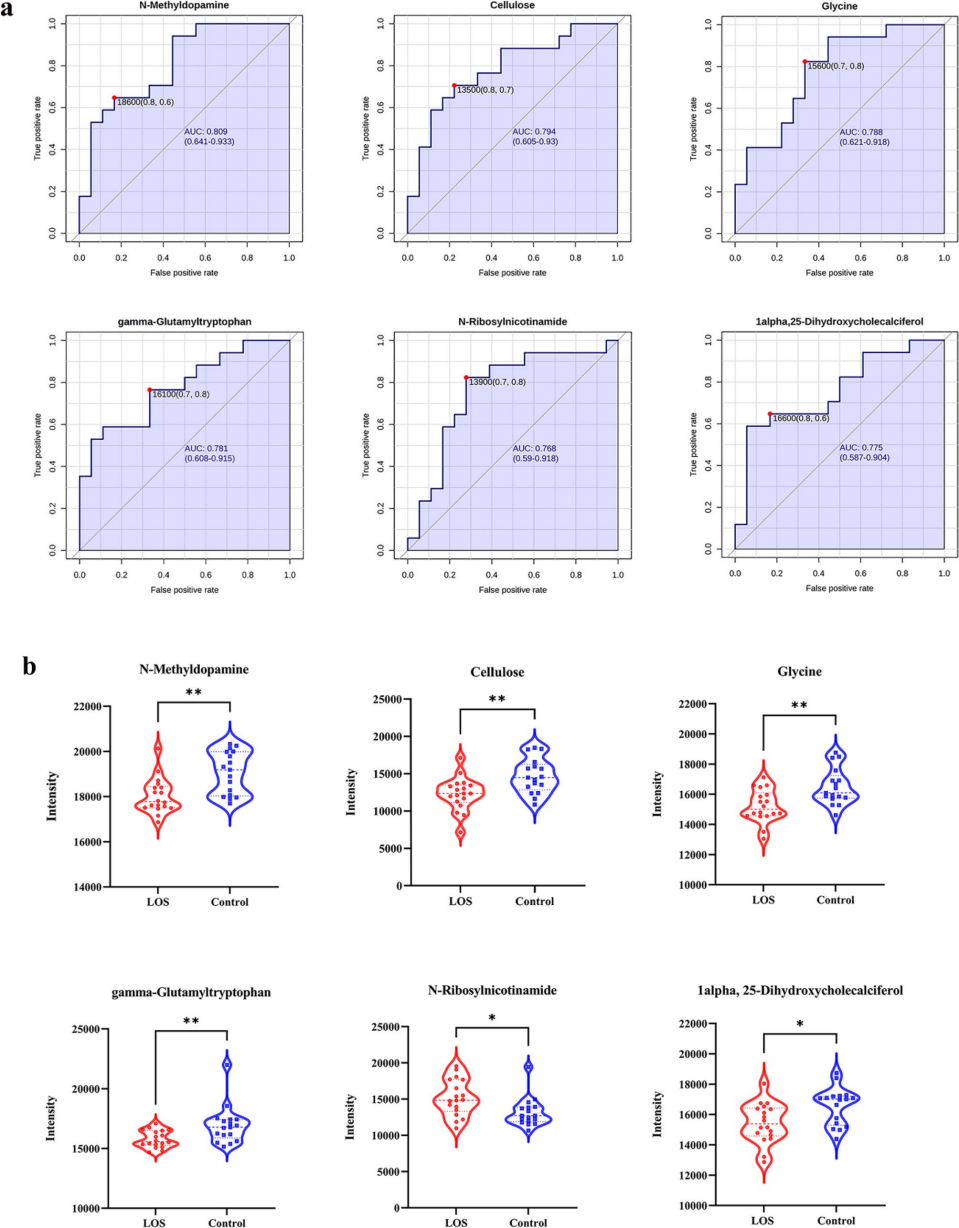
Comparison of ROC curves and intensity of differential metabolites. (**a**) Differential metabolite ROC curve and Cut-off value. (**b**) Comparison of different metabolite intensities between the LOS and control groups.

In our study, ROC curves, cut-off values and confusion matrices were calculated to evaluate the diagnostic value of differential metabolites for LOS in preterm infants (Fig. 5). N-Methyldopamine, cellulose, glycine, gamma-Glutamyltryptophan, N-Ribosylnicotinamide and 1alpha, 25-dihydroxycholecalciferol had the potential to diagnose LOS (AUC > 0.7).

### Pathway analysis

**Figure 6:**
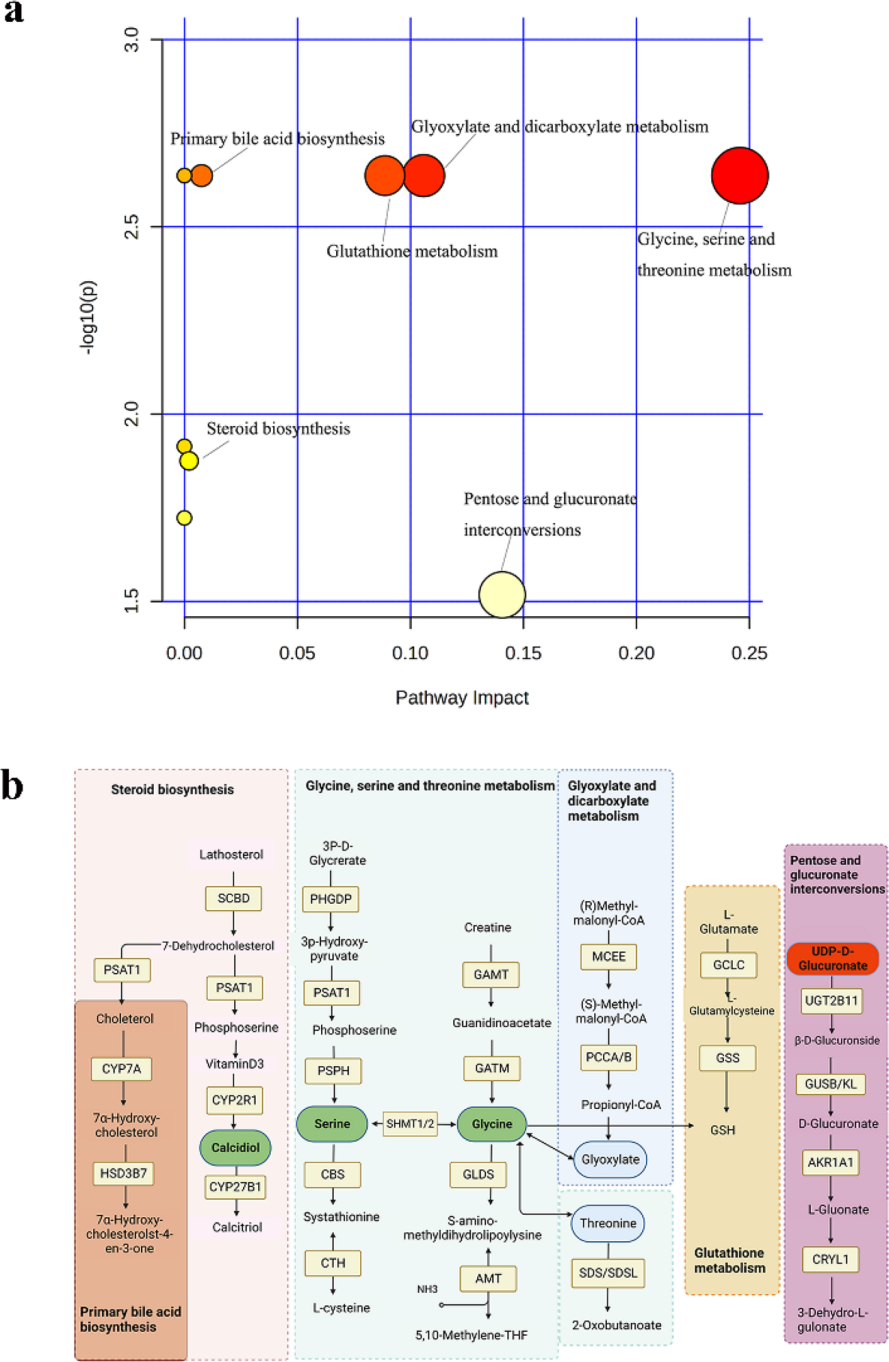
Metabolic pathway analysis. (**a**) Metabolic pathway map of differential metabolites. Each point represents the metabolic pathway. The size of the point is proportional to the impact value. The color of the point represents the P value of the metabolic pathway. (**b**) Schematic representation of metabolic pathways. Green indicates down-regulated metabolites, and red indicates up-regulated metabolites.

The differential metabolic pathways were analyzed by MetaboAnalyst 5.0 (https://www.metaboanalyst.ca) (Fig. 6a). Compared with KEGG database, KEGG value > 0.5 and *p* value < 0.05 were used as screening conditions for screening. Our study showed that glycine, serine, and threonine metabolism; glyoxylate and dicarboxylic acid metabolism; glutathione metabolism; primary bile acid biosynthesis; steroid synthesis; pentose and glucuronic acid interconversion may be involved in the pathogenesis of LOS. The pathway most likely involved is the metabolism of glycine, serine, and threonine. An elaborate representation of the pathways can be viewed in Fig. 6b.

## Discussion

Despite medical improvements, newborn sepsis remains a challenging problem in neonatology. Variations in GA and BW, age of onset, and types of bacteria causing the disease all contribute to the high incidence of LOS in hospitalized newborns. Early recognition of LOS can assist minimize sepsis-related mortality^16^. We investigated the characteristics of intestinal metabolites in preterm infants with LOS from a metabolomic perspective. We analyzed the differences in metabolites between infected and healthy infants to search for non-invasive biomarkers to aid in the early detection and diagnosis of LOS.

N-Methyldopamine, a metabolite of adrenaline that binds to dopamine and adrenergic receptors to activate adrenergic receptors and increase cellular metabolism, has been detected in prostate cancer cells^17^. Our study showed a significant decrease in the concentration of the intestinal metabolite N-Methyldopamine in preterm infants with LOS, suggesting that it may play an important biological role in the pathogenesis of LOS in preterm infants. The concentration of cellulose analogs in the feces of LOS infants was significantly reduced, and investigations into the clinical applications and biological effects of cellulose analogs have been reported previously. Positive therapeutic effects of hydroxypropyl cellulose were observed in DSS-induced acute colitis in mice^18^. Dietary cellulose may be protective against intestinal inflammation through regulation of lipid metabolism and gut microbiota ^19^. Gamma-Glutamyltryptophan has properties that boost the immune system, eliminate microbes, decrease inflammation, and inhibit tumor growth. This new peptide helps regulate the immune system and has shown promise in reducing radiation-related mouth sores in animals^20^. It could be a potential treatment for recurrent genital herpes simplex virus type 2^21^. In this study, the expression of gamma-Glutamyltryptophan was significantly decreased in the stool of premature infants with LOS, which may be a biomarker for early diagnosis of LOS and a potential bioactive for early intervention. 1alpha, 25-dihydroxycholecalciferol or cholecalciferol is vitamin D and its derivatives. Vitamin D deficiency is associated with cardiovascular, autoimmune, and infectious diseases^22^. Results from a cohort study of vitamin D levels and LOS in preterm infants showed that neonates with higher vitamin D levels had a lower risk of LOS than those with vitamin D deficiency, neonatal and maternal vitamin D deficiency increased the risk of LOS, and neonatal vitamin D was an independent risk factor for LOS^23^. According to our study, fecal 1alpha, 25-dihydroxycholecalciferol levels were significantly reduced in preterm infants with LOS. Intestinal 1alpha, 25-dihydroxycholecalciferol may be a sensitive and non-invasive biomarker for LOS.

Glycine exhibits anti-inflammatory properties, modulates immunological responses, and provides protection against oxidative stress^24^. An animal study suggests a link between low urinary glycine levels and intestinal dysfunction in piglets born prematurely^25^. According to clinical research, the activation of glycine receptors by glycine and serine has been found to effectively reduce myocardial inflammation. Additionally, this activation indirectly inhibits collagen formation in cardiac fibroblasts, leading to the eventual inhibition of myocardial fibrosis^26^. In addition, glycine mitigates intestinal damage by maintaining the mTOR pathway and inhibiting AMPK, TLR4, and NOD signaling, restores intestinal mucosal integrity after LPS-induced injury, and is directly utilized in the intestine for protein synthesis^27^. The findings of our study indicate a notable reduction in fecal glycine concentration among preterm infants diagnosed with LOS. It indicates that monitoring fecal glycine concentration could potentially serve as a promising method of early identification for the onset of LOS in preterm infants.

Finally, we analyzed the metabolic pathways of intestinal metabolites in LOS. Six pathways were significantly enriched, among which glycine, serine, and threonine pathways were most likely involved in the pathogenesis of LOS in preterm infants. The evidence from adult studies that glycine, serine, and threonine metabolism may play a role in the immunopathogenesis of the disease^28^. Furthermore, prior research have demonstrated that the metabolism of glycine, serine, and threonine is stimulated during the development of malignant tumors^29^. Glyoxylate and dicarboxylate metabolism bypass the TCA cycle, participate in energy metabolism, and are important in fungal infections^30^. Glutathione metabolism is involved in biological processes such as ferroptosis and antioxidation^31^, and glutathione is a major antioxidant that maintains redox homeostasis, which is closely associated with inflammatory responses^32^. Steroid hormone biosynthesis and primary bile acid biosynthesis involved in pathogenesis of acute and chronic infections in mice^33^. Some clinical studies have shown that patients with sepsis have a different characteristic steroid distribution than healthy subjects, with a marked decrease in steroidogenesis and in the adrenocorticotropic hormone-stimulated cortisol-corticosterone ratio, which predicts the risk of in-hospital death^34^. Animal studies suggest that pathways such as pentose glucuronic acid interconversion, pentose phosphate pathway, and galactose metabolism are involved in the metabolism of fecal microbiota^35^. The results of the present study regarding the metabolic pathways are consistent with the results of the above studies.

This study had several limitations. Firstly, this study lacked fecal samples from late preterm and term infants for metabolomics. Secondly, our study could not obtain maternal fecal samples to detect the association of fecal metabolites when comparing mothers and infants with LOS. Finally, given that prematurity and several unavoidable reasons limit breastfeeding for almost all infants with LOS, it would have been more meaningful to investigate the differences between breastfeeding and formula feeding on the metabolic process of LOS.

## Conclusion

Our study combined ROC curve analysis with fecal metabolite analysis of preterm infants with LOS. We screened the differential metabolites and pathways that are informative for the diagnosis of LOS. We found that Glycine, serine, and threonine metabolism; glyoxylate and dicarboxylic acid metabolism; glutathione metabolism; primary bile acid biosynthesis; steroid synthesis; pentose and glucuronic acid interconversion may be involved in the pathogenesis of LOS in preterm infants. The detection of changes in N-methyldopamine, cellulose, glycine, gamma-glutamyltryptophan, N-Ribosylnicotinamide and 1-alpha, 25-dihydroxycholecalciferol in preterm feces may be valuable in the early diagnosis of LOS. The metabolic pathway and metabolites may provide a promising and non-invasive method for the diagnosis and treatment of sepsis. The mechanisms of the metabolism and signaling in the sepsis condition remain completely clear which is the focus of our ongoing advanced study.

### Supplementary Information

Supplementary Information 1.

Supplementary Information 2.

## Data Availability

All data generated or analyzed during this study are included in this published article [and its supplementary information files].
