# Supplementary material for: Intestinal metabolomics in premature infants with late-onset sepsis
Source: Sci Rep. 2024 Feb 26;14:4659. doi: 10.1038/s41598-024-55398-7 (PMC10897474; doi:10.1038/s41598-024-55398-7)
Supplement: Supplementary file 2 — Supplementary Information 2. [file 41598_2024_55398_MOESM2_ESM.docx]

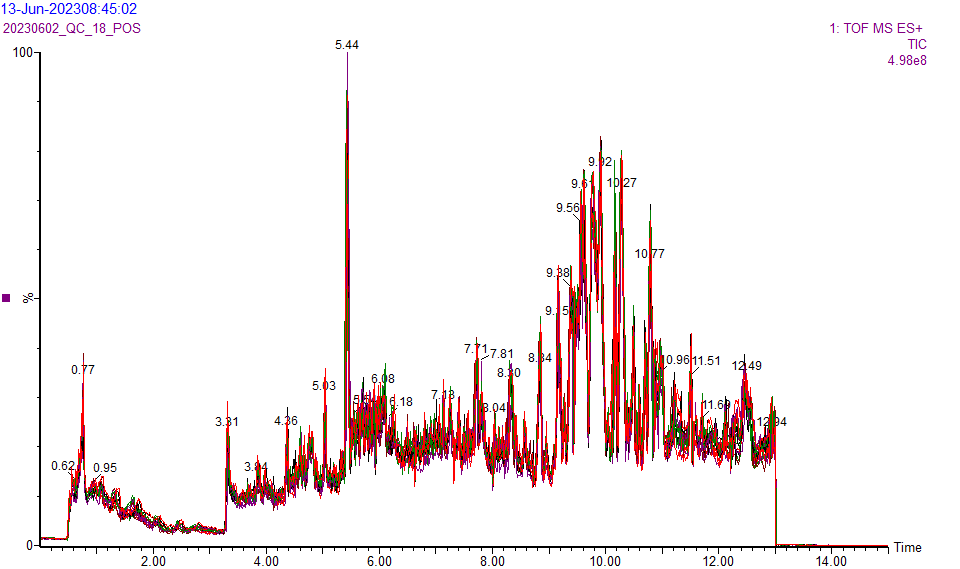


**Figure S1.** Data quality control. The overlapping TIC maps for the 13 QC samples, all QC samples in the figure have good overlap maps, and the retention time of each QC sample and its corresponding peak fluctuations were small, indicating that the detection instrument performs well during sample detection and analysis.
